# The Hidden Side of Complement Regulator C4BP: Dissection and Evaluation of Its Immunomodulatory Activity

**DOI:** 10.3389/fimmu.2022.883743

**Published:** 2022-04-25

**Authors:** Inmaculada Serrano, Ana Luque, Francesca Mitjavila, Anna M. Blom, Santiago Rodríguez de Córdoba, M. Cristina Vega, Joan Torras, Josep M. Aran

**Affiliations:** ^1^ Immune-inflammatory Processes and Gene Therapeutics Group, Institut d'Investigació Biomèdica de Bellvitge (IDIBELL), L’Hospitalet de Llobregat, Barcelona, Spain; ^2^ Internal Medicine Service, Bellvitge University Hospital, University of Barcelona and Institut d'Investigació Biomèdica de Bellvitge (IDIBELL), L’Hospitalet de Llobregat, Barcelona, Spain; ^3^ Department of Translational Medicine, Section of Medical Protein Chemistry, Lund University, Malmö, Sweden; ^4^ Molecular Pathology/Genetics of Complement Group, Centro de Investigaciones Biológicas Margarita Salas (CSIC) and Ciber de Enfermedades Raras (CIBERER), Madrid, Spain; ^5^ Structural Biology of Host-Pathogen Interactions Group, Centro de Investigaciones Biológicas Margarita Salas (CSIC), Madrid, Spain; ^6^ Nephrology Department, Bellvitge University Hospital, Experimental Nephrology Lab., University of Barcelona and Institut d'Investigació Biomèdica de Bellvitge (IDIBELL), L’Hospitalet de Llobregat, Barcelona, Spain

**Keywords:** PRP6-HO7, lupus nephritis, dendritic cells, inflammation, immunomodulation, C4BP(β-)

## Abstract

C4b-binding protein (C4BP) is a well-known regulator of the complement system that holds additional and important activities unrelated to complement inhibition. Recently, we have described a novel immunomodulatory activity in the minor C4BP(β-) isoform directly acting over inflammatory phagocytes. Here we show that incorporation of the β-chain to the C4BP α-chain oligomer interferes with this immunomodulatory activity of C4BP. Moreover, an oligomeric form including only the complement control protein 6 (CCP6) domain of the C4BP α-chain (PRP6-HO7) is sufficient to “reprogram” monocyte-derived DCs (Mo-DCs) from a pro-inflammatory and immunogenic phenotype to an anti-inflammatory and tolerogenic state. PRP6-HO7 lacks complement regulatory activity but retains full immunomodulatory activity over inflammatory Mo-DCs induced by TLRs, characterized by downregulation of relevant surface markers such as CD83, HLA-DR, co-stimulatory molecules such as CD86, CD80 and CD40, and pro-inflammatory cytokines such as IL-12 and TNF-α. Furthermore, PRP6-HO7-treated Mo-DCs shows increased endocytosis, significantly reduced CCR7 expression and CCL21-mediated chemotaxis, and prevents T cell alloproliferation. Finally, PRP6-HO7 shows also full immunomodulatory activity over Mo-DCs isolated from lupus nephritis patients with active disease, even without further pro-inflammatory stimulation. Therefore PRP6-HO7, retaining the immunomodulatory activity of C4BP(β-) and lacking its complement regulatory activity, might represent a promising and novel alternative to treat autoimmune diseases.

## Introduction

The complement system is an essential component of innate immunity. This evolutionarily conserved effector system, in addition to its crucial function in the innate defense against common pathogens, also holds a key regulatory and anti-inflammatory role in the “silent” clearance of immune complexes from the circulation and dying cells from damaged tissues, in close crosstalk with the mononuclear phagocyte system (1, 2). The complement cascade is tightly regulated by multiple regulatory proteins, as inappropriate activation is known to result in inflammation and host tissue destruction (3). The major proteins that regulate complement activation, both cell surface and fluid phase inhibitors, belong to the regulators of complement activation (RCA) family, and are exclusively composed of complement control protein (CCP) modules (4).

C4b-binding protein (C4BP) is the primary fluid phase inhibitor of the classical and lectin pathways of complement activation. It is synthesized mainly in the liver and is present in the circulation primarily as a major heterooligomeric isoform composed of seven α-chains and one β-chain termed C4BP(α7β1) or C4BP(β+), and a minor homooligomeric isoform preserving the seven α-chains but lacking the β-chain, dubbed C4BP(α7β0) or C4BP(β-). The α- and β-chains from both C4BP isoforms are held together in their C-terminus through an oligomerization domain which determines the spatial alignment of the chains, forming a spider-like structure. Both chains are, in turn, composed of a linear array of complement control protein (CCP) or “sushi” domains (α-chain (70 kDa): eight CCP domains; β-chain (40 kDa): three CCP domains) (5, 6). The β-chain binds non-covalently yet with high affinity (0.1 nM) to vitamin K-dependent anticoagulant protein S (PS) through its N-terminal CCP1 domain. Thus, under physiologic conditions the major C4BP(β+) isoform is joined to PS as C4BP(β+)-PS. This 570 kDa bimolecular complex endows additional roles in coagulation and in apoptotic/necrotic cell binding (7–9). Conversely, C4BP(β-) does not bind PS. Interestingly, under strong pro-inflammatory conditions (e.g., acute phase), the presence of circulating C4BP(β-) isoform increases, inducing a change in the C4BP(β+):C4BP(β-) ratio from ~ 80:20 to ~ 50:50 (10, 11). Nevertheless, both isoforms are equally competent for complement inhibition, which involves the N-terminal CCP1-CCP3 domains. These N-terminal domains bind several ligands, including the complement proteins C3b and C4b, heparin, pentraxins, CD91, DNA, and surface proteins from several bacterial pathogens (12).

Additionally, we have discovered a novel non-canonical activity in the minor isoform of C4BP, C4BP(β-). CCP domain-deletion mutagenesis in C4BP(β-) has revealed that CCP6 is required for this anti-inflammatory and tolerogenic activity by direct interaction with monocyte-derived dendritic cells (Mo-DCs), an established model of inflammatory DCs (13).

In this report we demonstrate that C4BP(β-), a modular RCA protein in structure, is also provided of functional modularity by displaying a dual activity. Thus, while the CCP1-CCP3 domains are required for the canonical complement inhibitory activity of C4BP(β-), the CCP6 domain is responsible for its non-canonical anti-inflammatory and tolerogenic activity. In contrast C4BP(β+), even whether devoid of PS, lacks the non-canonical activity. Moreover, we show that the CCP6 domain is not only necessary but also sufficient, whether oligomerized, for the immunomodulatory activity of C4BP(β-). PRP6-HO7, a recombinant heptamer resulting by joining CCP6 and the oligomerization domains of C4BP, lacks the complement inhibitory activity of C4BP while fully preserving its immunomodulatory activity. We recently confirmed the therapeutic potential of the non-canonical activity of C4BP(β-) to limit the development of lupus nephritis (LN) in two different animal models (14). We reveal here that PRP6-HO7 reverses the pro-inflammatory phenotype of monocytes isolated from LN patients, which supports its therapeutic potential in autoimmune pathologies.

## Materials And Methods

### Obtention and Purification of C4BP(β+), C4BP(β-), PRP5/8-HO7, PRP6-HO7 and PRP6-NO

As previously described, the C4BP(β+) and C4BP(β-) isoforms were differentially purified from pooled human plasma supplied by the local blood bank through BaCl_2_ precipitation (13). An alternative form of C4BP(β+) devoid of the anticoagulant PS, dubbed “C4BP(β+) naked”, was obtained by dialyzing C4BP(β+) (C4BP(β+)-PS) against 10 mM Tris-HCl (pH 7.5) containing 80% ethylene glycol plus heparin-sepharose chromatography.

PRP6-HO7 (Proline-rich Protein domain CCP6 heptamerized) is a 100 kDa homooligomer of seven recombinant polypeptides (14.3 kDa/each) engineered by fusing the N-terminal 6xHis-tagged CCP6 domain and the oligomerization domain of the C4BP α-chain. Analogously, in PRP6-NO (Proline-rich Protein domain CCP6 not oligomerized) (12.7 kDa), the C4BP α-chain has been engineered by fusing its N-terminal 6xHis-tagged CCP6 domain and a mutated version of the C4BP oligomerization domain unable to oligomerize by replacement of two cysteine residues by alanine and by deletion of its last 13 C-terminal amino acids (Δ537-549/C498A/C510A) (15).

The α-chain sequences corresponding to recombinant full-length C4BP(β-) and variants PRP5/8-HO7 (Proline-rich Protein domains CCP5-CCP8 heptamerized), PRP6-HO7, and PRP6-NO were all cloned into the pCDNA3.1(+) expression vector (ThermoFisher, Waltham, MA). Plasmid DNAs were amplified and purified using a Qiafilter Plasmid MegaKit (Qiagen, Hilden, Germany). Expi293 cells (ThermoFisher) were grown in suspension cultures until reaching the desired volume at a cell density of 2.5x10^6^ cells/ml and 98% viability. Cells were transiently transfected with 1 mg DNA per liter of culture and incubated for 7 days at 37°C, shaking at 125 rpm, with 8% CO_2_ supply. Finally, the culture media was collected and centrifuged for 30 min, 6000g at 4°C. The cell culture supernatants were used for protein purification. Recombinant full-length C4BP(β-) and variant PRP5/8-HO7 purification included four sequential chromatography steps: heparin chromatography, hydrophobic interaction (butyl) chromatography, anion exchange (Q Sepharose) chromatography and, finally, size exclusion (Superdex) chromatography. PRP6-HO7 and PRP6-NO were purified by nickel affinity chromatography (HisTrap FF) according to standard procedures. All chromatography columns were from GE Healthcare Bio-Sciences AB (Uppsala, Sweden). The proteins were concentrated, dialyzed, and recovered in PBS buffer, pH 7.4, and their purity was higher than 85%, as assessed by Bis-Tris 4-12% SDS-PAGE (NuPAGE precast protein gels; ThermoFisher) and further Blue Safe staining (NZYTech, Lisboa, Portugal).

### PRP6-HO7 Structure Prediction

The homooligomeric structure of PRP6-HO7 was predicted by comparative protein structure modeling with MODELLER v10.1 (16) using suitable crystallographic structures as multiple templates. The structure of the C4BP α-chain CCP1 and CCP2 domains (PDB 5I0Q) were used as templates for the N-terminal CCP6 domain of PRP6-HO7 (17) and the C-terminal heptameric core complex structure of C4BP was taken from PDB 4B0F (18). Suitable restrains were introduced to ensure the proper recognition of the two disulphide bonds present in CCP6 (Cys365-Cys409 and Cys399-Cys422), and the heptameric organization of the C-terminal helical motif was stabilized through symmetry restraints. One thousand models were calculated for the target sequence and the model with the lowest value for the DOPE assessment score was selected as the best model (19). The stereochemical quality of the selected model was further evaluated with MolProbity (20).

### Monocyte Culture and C4BP-Derived Protein Treatment

Total blood from healthy donors was acquired in the Blood and Tissue Bank (Barcelona, Spain) and their PBMCs were isolated at less than 16 h after extraction through Ficoll-Paque density centrifugation (GE Healthcare Bio-Sciences AB).

Systemic lupus erythematosus (SLE) patients derived to the Nephrology and Internal Medicine Units (UFMAS) from Bellvitge University Hospital underwent blood extraction at hospital admission, and their PBMCs were obtained as described for healthy donors. This study was approved by the IDIBELL’s ethics committee in accordance with institutional guidelines and the Declaration of Helsinki, and the patients’ written informed consent was obtained.

For all functional assays, monocytes were purified using colloidal super-paramagnetic microbeads conjugated with monoclonal mouse anti-human CD14 antibodies (MACS, Miltenyi Biotec, Auburn, CA) and counted using Perfect Count microspheres (Cytognos SL, Salamanca, Spain). In addition, the purity of CD14+ cells was tested by CD14 staining and flow cytometry analysis (FACSCanto II flow cytometer equipped with FACSDiva software (Becton Dickinson, Franklin Lakes, NJ)), allowing the assessment of the number of PBMCs and the number of total CD14+ cells (>90% CD14+).

Monocytes were plated at 2.5x10^5^ cells/500 µl in 24 well culture plates (Jet Biofil, Guangzhou, China), in RPMI 1640 (Gibco, ThermoFisher) supplemented with 100 mg/ml streptomycin, 100 IU/ml penicillin, 2 mM L-glutamine (all from Invitrogen, Carlsbad, CA) and 10% heat-inactivated FBS (Life technologies, ThermoFisher) (complete medium) at 37 °C under 5% CO_2_. Monocyte-derived DCs (Mo-DCs) or monocyte-derived macrophages (Mo-macrophages) were generated supplementing the monocyte cultures with complete RPMI 1640 medium plus GM-CSF (800 IU/ml) and IL-4 (500 IU/ml) (Mo-DCs) or GM-CSF (650 IU/ml) (Mo-macrophages) both from Gentaur, Kampenhout, Belgium.

C4BP(β+), C4BP(β-), and PRP variants were added at day 0 to differentiating monocytes at the indicated concentrations. For DC maturation, Mo-DCs, either untreated or treated with these C4BP-derived molecules, were further stimulated for 48 h with 5 µg/ml LPS (*Escherichia coli* 055.B5, Sigma Aldrich, Merck, Darmstadt, Germany) or 10 µg/ml Gardiquimod (Imidazoquinoline compound; TLR7 ligand) (Invivogen, San Diego, CA) at day 5.

Moreover, to assess the influence of human serum in C4BP(β-) and PRP6-HO7 immunomodulatory activity, human Mo-DCs were treated with the respective PRP variants at the indicated concentrations and co-incubated with 50% of heat-inactivated human serum (56°C, 1h) through their differentiation and maturation process.

### TLR Activation

Maturation of Mo-DCs was induced by 48 h incubation with a panel of TLR agonists: Pam3CSK4 (TLR1/2) (300 ng/ml), HKLM (Heat-killed *Listeria monocytogenes*) (TLR2) (10^8^ cells/ml), Poly HMW (I:C) and Poly LMW (I:C) (TLR3) (10 μg/ml/each), LPS-EK (LPS *E.coli K12*) (TLR4) (5 μg/ml), FLA-ST (Flagellin *S. typhimurium*) (TLR5) (2 μg/ml), FSL1 (TLR6/2) (100 ng/ml), Gardiquimod (TLR7) (10 μg/ml), ssRNA40/LyoVec (TLR8) (5 μg/ml), E. coli ssDNA/LyoVec (TLR9) (10 μg/ml) (all from Invivogen).

### Abs and Flow Cytometry

Cell-surface phenotypes were analyzed using the following MoAbs: APC-conjugated anti–CD64 (10.1.1), FITC-conjugated anti-CD14 (TÜK4), PE-conjugated anti-CD40 (HB14), PE-conjugated anti-CD86 (FM95), FITC-conjugated HLA-DR (REA805), APC-conjugated anti-CD83 (REA714), PE-conjugated anti-CD80 (REA661) (Miltenyi Biotec), FITC-conjugated anti-CD3 (UCHT1) (Tonbo Biosciences, San Diego, CA) and Alexa Fluor 488-conjugated anti-CCR7 (G043H7) (Biolegend, San Diego, CA). APC-conjugated anti-IgG1 (IS5-21F5), FITC-conjugated anti-IgG2a (S43.10), PE-conjugated anti-IgG1 (IS5-21F5), REA control antibody (S) human IgG1 (Miltenyi Biotec), FITC-conjugated anti-IgG1κ (MOPC-21) (Tonbo Biosciences), and Alexa Fluor 488-conjugated anti-IgG2aκ (MOPC-173) (Biolegend) were used as the respective isotype controls.

After washing with PBS, cells were subsequently stained with the respective MoAbs, according to the manufacturer’s instructions, in 100 ml FACS buffer (PBS containing 1% BSA and 0.1% sodium azide) for 15 min at room temperature. We gated the cells according to forward scatter (FSC) and side scatter (SSC) parameters to exclude debris. Staining with 7-aminoactinomycin D (ThermoFisher) was also employed to assess their viability status. Stained cells were analyzed using a FACSCanto II flow cytometer (Becton Dickinson). Subsequent analyses used FlowJo software (Flowjo LLC, Ashland, OR).

### Endocytic Activity

To measure endocytosis of Mo-DCs, 2x10^5^ cells/ml were resuspended in 60 μl of complete medium and incubated with 4 μl of BODIPY FL-conjugated DQ-Ovalbumin (1 mg/ml, DQ-OVA, Molecular Probes, Leiden, Netherlands) at 37°C or at 0 °C for 30 min (receptor-dependent endocytosis). The incubations were stopped by adding 150 μl of cold FACS buffer. The cells were washed two times with cold FACS buffer, and their fluorescence was analyzed using flow cytometry.

### Chemotaxis

Mo-DCs differentiated in presence of C4BP(β+), C4BP(β-), or PRP6-HO7, and matured (LPS for 48 h), were tested for migration toward the CCL21 chemokine using transwell assays. Briefly, the lower chambers of transwell plates (polycarbonate filters of 8.0 μm pore size; Costar, Corning, NY) were filled with 400 μl of complete RPMI 1640 medium with or without CCL21 (200 ng/ml). A total of 1.6x10^4^ DCs in 100 μl of complete RPMI 1640 medium were added into the upper chamber, and cells were incubated at 37°C for 2 h. Cells migrated into the lower chambers were harvested and counted with a flow cytometer, acquiring events for a fixed time period of 1.5 min. The migration assays for all stimulation conditions were performed in duplicate wells. Values are given as total number of migrated cells.

### Mixed Leukocyte Reaction

For the co-culture assays, CD3^+^ T cells were isolated from PBMCs by negative selection using MojoSort™ Human CD3 T Cell Isolation Kit (Biolegend). CD3^+^ T cells were 90% pure, as assessed by CD3 staining and flow cytometry. Allogeneic CD3^+^ T cells were labeled with the intracellular fluorescent dye CFSE (CellTrace™) (Invitrogen, ThermoFisher) according to the manufacturer’s recommendations, cultured in 96-well round-bottom plates in RPMI complete medium at a density of 1x10^5^ cells/well, and stimulated for 5 days with C4BP(β+)-, C4BP(β-)- or PRP6-HO7-treated and LPS-activated DCs at 1:5 DC:T cell ratio. CD3^+^ T cell proliferation was determined by sequential loss of CFSE fluorescence and quantified by flow cytometry.

### Intracellular Cytokine Staining

Total cells from the co-culture assays were stimulated with 50 ng/ml PMA plus 500 ng/ml ionomycin for 5 h in the presence of 10 μg/ml brefeldin A (all from Sigma Aldrich). After stimulation, cells were washed with PBS, fixed and permeabilized using an IntraStain kit (Dako, Agilent, Santa Clara, CA), and incubated for 30 min at room temperature with APC-conjugated anti-human IFN-γ MoAb (45–15) (Miltenyi). Finally, cells were washed and analyzed by flow cytometry.

### DC Cytokine Secretion

Concentrations of human IL-12p70, TNF-α, and IL-10 were determined from DC supernatants treated with the C4BP isoforms or PRP-based proteins using the respective DuoSet ELISA kits (R&D Systems, Minneapolis, MN) according to the manufacturer’s instructions.

### SDS-PAGE and Western Blot Analysis

C4BP isoforms and PRP variants were resolved on 4-12% gradient SDS-PAGE (NuPAGE Bis-Tris 4-12% Mini Gels, Invitrogen) under reducing or non-reducing conditions, and either immersed in Blue Safe (NZYTech) for 15 min and destained in water or transferred to a PVDF membrane for Western blot analysis. After blocking, the membrane was probed overnight at 4 °C with primary antibodies: 1:2000 dil. polyclonal PK9008 rabbit anti-C4BP α-chain (8) and 1:4000 dil. monoclonal anti-6xHis tag antibody (Takara Bio, Mountain View, CA), and for 1 h at room temp for 1:1000 dil. monoclonal anti-human Protein S/PROS1 antibody (R&D Systems), followed by the addition of 1:2000 dil. polyclonal Goat Anti-Rabbit IgG HRP (P0448), and 1:2000 dil. polyclonal Goat Anti-Mouse IgG HRP (P0447) (both from Dako), respectively, for 1 h at room temp. Detection was performed with enhanced chemiluminescence (ECL) (Pierce, ThermoFisher) using ChemiDoc Imager (Bio-Rad, Hercules, CA).

### C4b Cofactor Activity Assay

Complement C4b (8.9 µg/ml) and factor I (4.4 µg/ml) (Merck, Darmstadt, Germany) were mixed with C4BP isoforms (0.6 nM or 6 nM) in low-salt buffer (25 mM phosphate buffer pH 7.4 and 25 mM NaCl) in a total volume of 60 µl and incubated at 37°C for 30 min. Then, reducing SDS sample buffer was added, C4b fragments were separated on a 4–12% gradient SDS-PAGE (NuPAGE Bis-Tris 4-12% Mini Gels, ThermoFisher), and Western blot analysis of C4b fragments was performed using a 1:2000 dilution of Anti-Human C4d MoAb (Quidel, San Diego, CA) overnight at 4 °C, followed by a 1:2000 dilution of polyclonal Goat Anti-Mouse IgG HRP (P0447, Dako) in TBS-Tween 0.05%, 1% BSA, 0.02% NaN_3_.

### Statistical Analysis

Statistical analyses and scientific graphing were performed using the GraphPad Prism 6 software (GraphPad software, Inc, La Jolla, CA). Repeated measures one-way ANOVA, corrected for multiple comparisons using Dunnett’s method was performed to contrast MFI, percentages of CSFE negative, and IFN-γ positive, T cells, and cytokine levels under different experimental conditions with respect to a reference condition. Results from migration assays with or without CCL21 were compared with two-way ANOVA and Dunnett’s test. To compare MFI and % of positive cells between two different conditions, the paired t-test was employed. The relationship between the clinical features and the Mo-DC and Mo-macrophage surface marker expression in SLE patients was assessed by Spearman’s rank correlation test. Data are expressed as mean values ± SD. In all cases, a P-value < 0.05 was considered significant.

## Results

### The β-Chain Interferes With the Immunomodulatory Activity of C4BP

**Figure 1:**
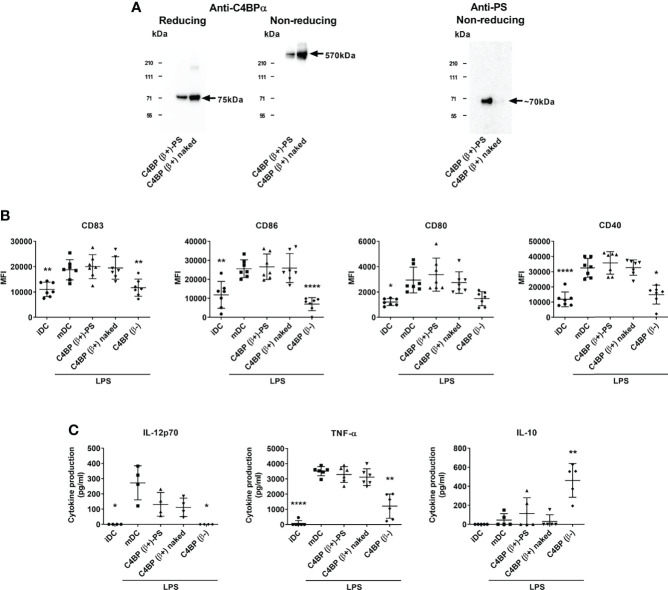
The β-chain of C4BP impairs the immunomodulatory activity of its internal CCP6 α-chain domain. **(A)** SDS-PAGE and Western analysis of the major physiological C4BP isoform (C4BP(β+)) complexed with PS (C4BP(β+)-PS) through its β-chain, and the same C4BP(β+) isoform devoid of PS (C4BP(β+) naked). Both proteins were resolved under reducing (left) and non-reducing (middle) conditions and probed with PK9008 anti-C4BP α-chain antibody. A further Western blot probed with an anti-PS antibody confirmed the absence of PS in the “C4BP(β+) naked” form (right). **(B)** Human Mo-DCs were incubated throughout their differentiation process with C4BP(β+)-PS, C4BP(β+) naked and C4BP(β-), all at 12 nM (corresponding to 6.0 μg/ml, 5.3 μg/ml, and 5.0 μg/ml, respectively). DC maturation was achieved by LPS treatment. Cells were then collected, washed, and analyzed by flow cytometry for cell surface expression of the activation marker CD83 and the co-stimulatory molecules CD86, CD80 and CD40. MFI, median fluorescence intensity for the different surface markers. **(C)** The concentrations of IL-12p70, TNF-α and IL-10 were analyzed in the respective cell supernatants by ELISA. iDC, untreated, immature DCs; mDC, untreated, LPS-matured DCs. The results shown are the mean ± SD from 7 independent donors (cell surface markers), or from 4-6 independent donors performed in duplicate (cytokines) (*p < 0.05; **p < 0.01; ****p < 0.0001 compared with mDC). IL-12p70 concentrations induced by C4BP(β+)-PS and C4BP(β+) naked appeared reduced but were not statistically significant compared to that induced by mDC (p = 0.082, and p = 0.053, respectively).

Among the main human C4BP isoforms only the minor C4BP(β-), but not the major C4BP(β+), displayed non-canonical anti-inflammatory and tolerogenic activity over Mo-DCs (13). Under physiological conditions, all β-chain-containing C4BP molecules in plasma are tightly bound to PS in a 1:1 stoichiometry (21, 22). Thus, the plasma-purified C4BP(β+) isoform is actually present as a high-affinity noncovalent C4BP(β+)-PS complex. While this complex does not interfere with the complement inhibitory activity of C4BP, held through the peripheral N-terminal CCP1-CCP3 domains of its α-chains, it does so for the non-canonical activity recognized in the CCP6 internal domain of its α-chains. Thus to assess which polypeptide, PS or the C4BP β-chain, was responsible for blocking the immunomodulatory activity in C4BP(β+), we excised PS from the purified C4BP(β+)-PS complex (23) and compared the functional consequences of incubating “C4BP(β+)-PS” and “C4BP(β+) naked” on Mo-DC differentiation/maturation. This particular assay was performed under serum-free conditions to avoid the recruitment of bovine PS by human “C4BP(β+) naked” because it has been shown that the human C4BP/bovine PS interaction has a 5-fold higher affinity than the human C4BP/human PS interaction (24). Western analysis confirmed that the purified “C4BP(β+) naked” isoform was indeed devoid of PS (**Figure 1A**). In contrast to C4BP(β-), neither “C4BP(β+)-PS” nor “C4BP(β+) naked” were able to significantly influence the overexpression of several LPS-induced Mo-DC activation markers, including the maturation marker CD83 and the co-stimulatory molecules CD86, CD80 and CD40 (**Figure 1B**). Analogously, only C4BP(β-), but not “C4BP(β+)-PS” or “C4BP(β+) naked”, was able to prevent the secretion of the pro-inflammatory cytokines IL-12 and TNF-α and, conversely, increased the production of the anti-inflammatory cytokine IL-10 (**Figure 1C**).

Taken together, these results demonstrate that incorporating the β-chain to the C4BP α-chain oligomer is responsible for the absence of immunomodulatory activity of the major C4BP(β+) isoform, and that this effect of the β-chain does not require the incorporation of the PS to the complex.

### C4BP Modularity Allows Dissection of Its Immunomodulatory Activity

**Figure 2:**
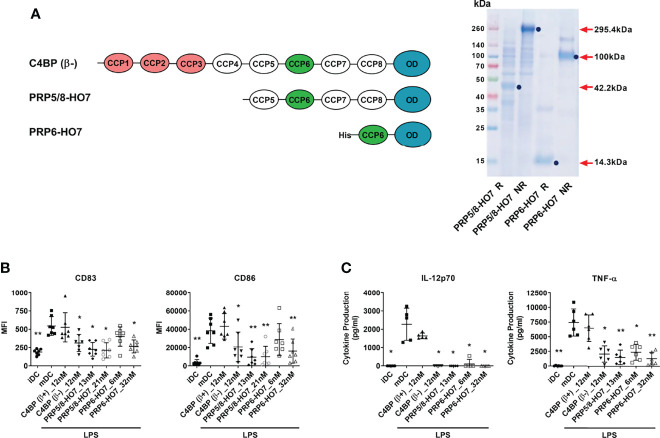
Deletion of the complement inhibitory domains of C4BP(β-) does not affect its immunomodulatory activity. **(A)** Left: Schematic structure of the α-chains from C4BP(β−), and from its variants PRP5/8-HO7 and PRP6-HO7. The CCP1-CCP3 complement inhibitory domains are depicted in red. The CCP6 immunomodulatory domain is depicted in green. The oligomerization domain (OD) is depicted in blue. “His” refers to a poly-histidine tag located at the N-terminus of PRP6-HO7. Right: SDS-PAGE and Coomassie Blue staining of both PRP5/8-HO7 and PRP6-HO7 under reducing (R) and non-reducing (NR) conditions. Red arrows and blue dots indicate the location and size of both reduced and non-reduced protein forms. Left lane, molecular weight marker. **(B)** Human Mo-DCs were incubated throughout their differentiation process with C4BP(β+), C4BP(β-) (both at 12 nM) and the variants PRP5/8-HO7 and PRP6-HO7 at the indicated concentrations. DC maturation was achieved by LPS treatment. Cells were then collected, washed, and analyzed by flow cytometry for cell surface expression of the activation marker CD83 and the co-stimulatory molecule CD86. MFI, median fluorescence intensity for the different surface markers. **(C)** The concentrations of IL-12p70 and TNF-α were analyzed in the respective cell supernatants by ELISA. iDC, untreated, immature DCs; mDC, untreated, LPS-matured DCs. The results shown are the mean ± SD from 7 independent donors (cell surface markers), or from 5-6 independent donors performed in duplicate (cytokines) (*p < 0.05; **p < 0.01 compared with mDC).

We sought to dissect the non-canonical immunomodulatory activity of C4BP(β-) from its canonical complement regulatory activity to confirm its functional independence. Thus, we designed and recombinantly produced two novel C4BP variants: PRP5/8-HO7 and PRP6-HO7, both lacking the three outermost CCP1-CCP3 domains required for complement inhibition (25). Thus, variant PRP5/8-HO7 is an heptamer containing a truncated version (CCP5-CCP8 plus C-terminal oligomerization domain) of the C4BP α-chain, and variant PRP6-HO7 is an heptamer including only the CCP6 domain and the C-terminal oligomerization domain of the C4BP α-chain (**Figure 2A**). In PRP5/8-HO7, only the CCP1-CCP4 N-terminal domains of the C4BP α-chain were deleted to prevent potential misfolding issues around CCP6 that could affect its immunomodulatory activity.

To comparatively assess the immunomodulatory activity of the full-length recombinant C4BP(β−) isoform and its deletion variants PRP5/8-HO7 and PRP6-HO7, we pre-incubated Mo-DCs with the indicated concentrations of each protein or the inactive C4BP(β+) isoform and further challenged these cells with the pro-inflammatory and maturation stimulus LPS. As previously published (13), recombinant C4BP(β-), but not C4BP(β+), was able to confer a semi-mature, anti-inflammatory phenotype to LPS-matured Mo-DCs. Interestingly, both C4BP(β-) variants also showed a comparable immunomodulatory activity, as confirmed by downregulation of the CD83 and CD86 surface markers and prevention of pro-inflammatory IL-12 and TNF-α production in LPS-stimulated Mo-DCs (**Figures 2B, C**). Therefore, both PRP5/8-HO7 and PRP6-HO7 variants retained the full immunomodulatory activity ascribed to C4BP(β-), and PRP6-HO7 was selected for further characterization.

### Oligomerization is Necessary to Preserve the Immunomodulatory Activity of PRP6-HO7

**Figure 3:**
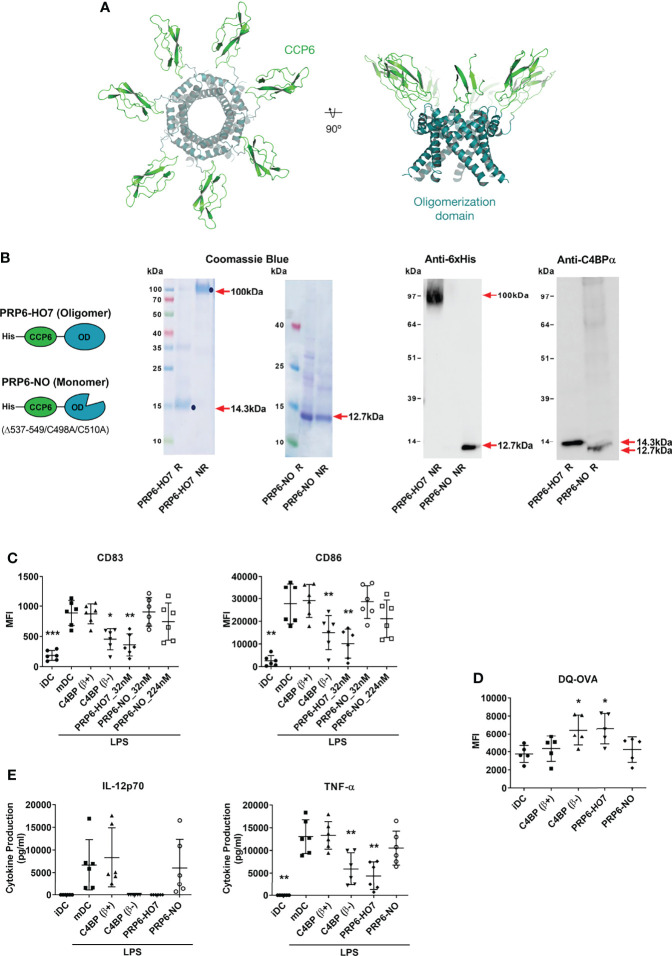
Oligomerization is necessary to preserve the immunomodulatory activity of the C4BP α-chain CCP6 domain. **(A)** Molecular modeling of PRP6-HO7. PRP6-HO7 homo-oligomer structure prediction by comparative protein structure modeling with MODELLER. PRP6-HO7 is shown in cartoon representation. The N-terminal CCP6 domain and the C-terminal oligomerization domain are shown in green and blue, respectively. **(B)** Schematic structure of the monomer chain from the PRP6-HO7 heptamer, and from PRP6-NO, unable to oligomerize because of a mutated/truncated OD. PRP6-HO7 and PRP6-NO were visualized by SDS-PAGE and Coomassie Blue staining, and by Western analysis against anti-His and anti-C4BP α-chain antibodies, under both reducing (R) (PRP6-HO7 monomer, 14.3 kDa; PRP6-NO, 12.7 kDa) and non-reducing (NR) (PRP6-HO7 oligomer, 100 kDa; PRP6-NO, 12.7 kDa) conditions. Red arrows indicate the respective molecular weights. **(C)** Human Mo-DCs were incubated throughout their differentiation process with C4BP(β+), C4BP(β-) (both at 12 nM) and the variants PRP6-HO7 and PRP6-NO (both at 32 nM, unless otherwise stated). DC maturation was achieved by LPS treatment. Cells were then collected, washed, and analyzed by flow cytometry for cell surface expression of the activation marker CD83 and the co-stimulatory molecule CD86. MFI, median fluorescence intensity for the different surface markers. The results shown are the mean ± SD from 6 independent donors (*p < 0.05; **p < 0.01; ***p < 0.001 compared with mDC). **(D)** Comparative endocytic activity of Mo-DCs was also assessed by flow cytometry, measuring fluorescent DQ-OVA internalization (receptor-mediated endocytosis) at the differentiation stage, after treatment with C4BP(β+), C4BP(β-), PRP6-HO7, and PRP6-NO. Data shown are the mean MFI ± SD from 5 independent experiments (*p < 0.05 compared with iDC). **(E)** The concentrations of IL-12p70 and TNF-α were analyzed in the cell supernatants from **(C)**, except the PRP6-NO_224 nM sample, by ELISA. iDC, untreated, immature DCs; mDC, untreated, LPS-matured DCs. The results shown are the mean ± SD from 6 independent donors performed in duplicate (**p < 0.01 compared with mDC).

PRP6-HO7 has a predicted heptameric structure analogous to the physiologic C4BP(β-) isoform, according to a comparative protein structure modeling analysis performed taking into account previous available structural data from the CCP1 and CCP2 domains of the C4BP α-chain and the C-terminal heptameric core complex of C4BP (17, 18). This novel recombinant protein appears as a compact symmetrical unit composed of seven CCP6 domain chains joined in their C-terminus to the C4BP oligomerization domain, forming a non-glycosylated radial spider-like heptamer (**Figure 3A**).

To investigate whether oligomerization of the C4BP CCP6 immunomodulatory domain was indeed necessary to preserve its immunomodulatory activity, we compared the functional outcome of heptavalent PRP6-HO7 with that of an analogous variant, PRP6-NO, comprising the CCP6 domain and a mutated oligomerization domain engineered by substitution of two Cys residues (C498A/C510A) essential for correct folding plus the additional truncation of its 13 C-terminal amino acids (15). As expected and confirmed through PAGE and both Coomassie Blue staining and Western blot analysis under reducing and non-reducing conditions, PRP6-HO7 folded into a heptameric 100 kDa structure consisting of seven monomer chains of 14.3 kDa, which included the CCP6 domain and the C-terminal oligomerization domain of C4BP. On the other hand, PRP6-NO could not oligomerize under non-reducing conditions and remained as a single monomer of 12.7 kDa (**Figure 3B**).

Both C4BP(β+) and PRP6-NO had no effect on the expression of the DC activation marker CD83 and the DC co-stimulatory molecule CD86 on LPS-matured DCs. Conversely, PRP6-HO7, analogously to C4BP(β-), significantly down-regulated these markers (**Figure 3C**).

On the other hand, the antigen internalization capacity of DCs was assessed by flow cytometry of self-quenching DQ-OVA (mannose receptor-mediated endocytosis marker). Thus, the endocytic activity of iDCs was significantly increased by treatment with either C4BP(β-) or PRP6-HO7 but neither by monomeric PRP6-NO nor by the inactive molecule C4BP(β+) (**Figure 3D**).

We next assessed whether the effect of the different C4BP-derived proteins on the DC phenotype was accompanied by changes in their release of pro-inflammatory cytokines (IL-12 and TNF-α). Compared to untreated iDCs, secretion of each of these cytokines was up-regulated when iDCs were matured with LPS. Furthermore, DCs pre-treated with both C4BP(β+) and PRP6-NO secreted the same cytokine levels as untreated DCs upon maturation. In contrast, pre-treatment with both C4BP(β-) and PRP6-HO7 prevented the release of IL-12 and significantly decreased the release of TNF-α (**Figure 3E**). Thus, Th1 pro-inflammatory cytokine production upon LPS-mediated DC stimulation was significantly diminished by PRP6-HO7-treated DCs.

DCs treated with the different C4BP-derived molecules remained highly viable throughout the differentiation/maturation process, as assessed by annexin V/7-ADD staining, with less than 10% apoptotic cells evidenced at 48 h after LPS-mediated DC maturation (data not shown).

Together, these data are evidence that oligomeric PRP6-HO7, but not monomeric PRP6-NO, has the potential to modify pro-inflammatory DC differentiation/maturation towards an anti-inflammatory and tolerogenic phenotype.

### PRP6-HO7 Lacks Complement Inhibitory Activity

**Figure 4:**
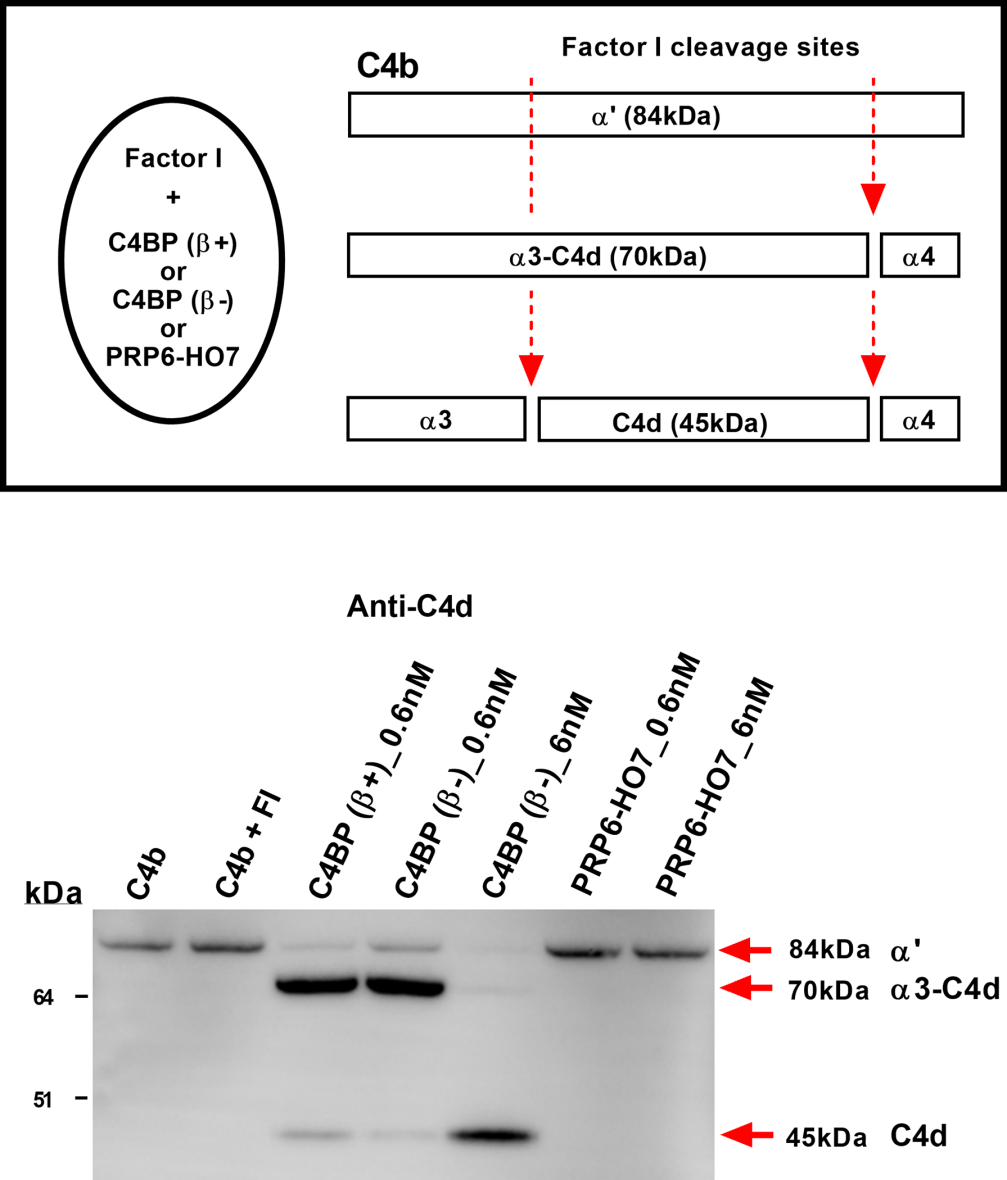
PRP6-HO7 is devoid of complement inhibitory activity. Schematic drawing of C4BP(β+) and C4BP(β-) cofactor activity for factor I-mediated splitting of C4b. Factor I cleaves the α′-chain of C4b at two sites. Partial cleavage generates fragments α3-C4d (70 kDa) and α4 (14 kDa). Further cleavage of α3-C4d yields the small C4d (45 kDa) fragment which remains associated with targets. C4BP(β+), C4BP(β-) and PRP6-HO7, at the indicated concentrations (lanes 3-7), were incubated with C4b (8.9 μg/ml) followed by addition of factor I (4.4 μg/ml). Reaction controls included C4b alone, and C4b + FI. All reactions were stopped after 30 min with SDS-reducing sample buffer. C4b cleavage fragments were separated by 4-12% SDS-PAGE under reducing conditions followed by Western blotting using an anti-C4d MoAb. Red arrows indicate the size of the C4b fragments. Cofactor activity was confirmed by the appearance of α3-C4d (70 kDa) or C4d (45 kDa). Results are representative of 3 independent experiments.

One of the major regulatory functions of C4BP is to serve as a cofactor for the serine protease factor I to inactivate C4b, also termed cofactor activity. To confirm that PRP6-HO7 was indeed devoid of complement inhibitory activity, allocated to the CCP1-CCP3 domains of the C4BP α-chain, we performed a comparative C4BP cofactor activity assay evaluating its contribution on factor I-mediated cleavage of C4b in solution. Thus, both C4BP(β+) and C4BP(β-), but not PRP6-HO7 at any of the concentrations tested, were able to act as a cofactor for factor I cleavage of the C4b α’-chain, yielding the 70 kDa partial cleavage fragment α3-C4d and the small fragment C4d (45 kDa) (**Figure 4**).

### PRP6-HO7 Attenuates TLR-Mediated Activation of Mo-DCs

**Figure 5:**
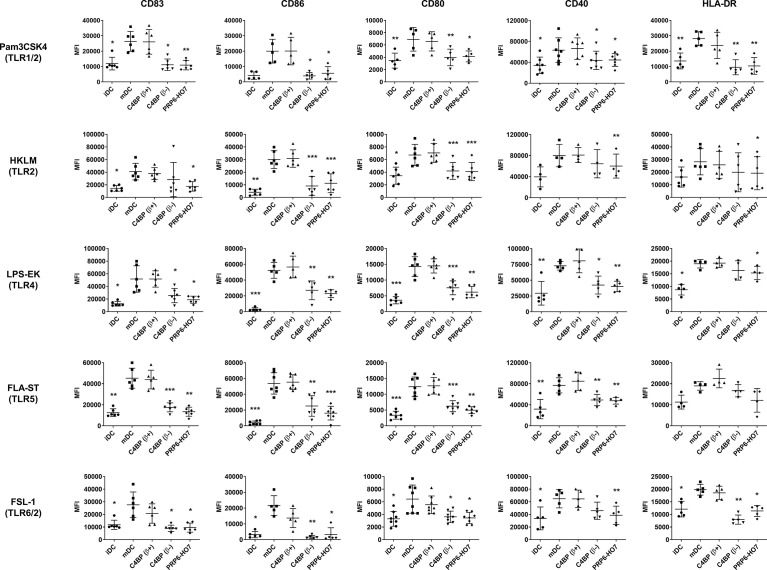
PRP6-HO7 prevents pro-inflammatory surface TLR activation of Mo-DCs. Human Mo-DCs were incubated throughout their differentiation process with C4BP(β+), C4BP(β-) (both at 12 nM) and PRP6-HO7 (32 nM). DC maturation was achieved by TLR agonist treatment: Pam3CSK4 (TLR1/2), HKLM (TLR2), LPS-EK (TLR4), FLA-ST (TLR5), FSL-1 (TLR6/2). Cells were then collected, washed, and analyzed by flow cytometry for cell surface expression of CD83, CD86, CD80, CD40 and HLA-DR. MFI, median fluorescence intensity for the different surface markers. iDC, untreated, immature DCs; mDC, untreated, TLR-matured DCs. The results shown are the mean ± SD from 4-8 independent donors (*, p < 0.05; **, p < 0.01; ***, p < 0.001 compared with mDC).

**Figure 6:**
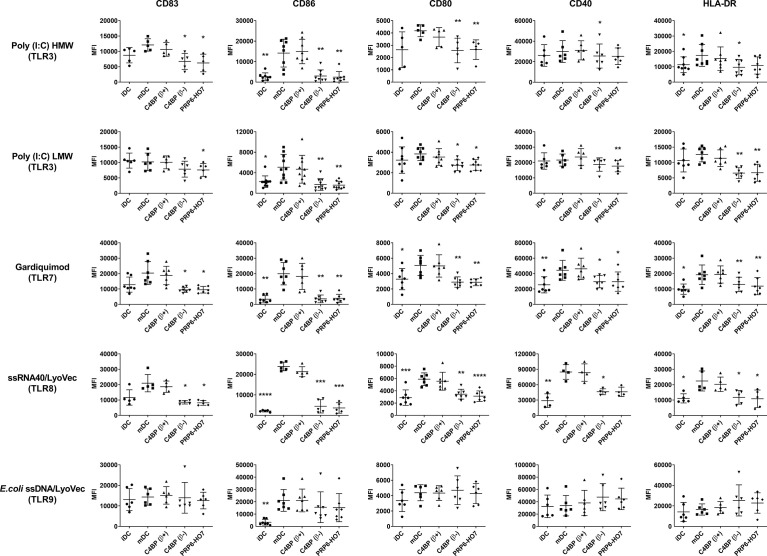
PRP6-HO7 prevents pro-inflammatory intracellular (endosomal) TLR activation of Mo-DCs. Human Mo-DCs were incubated throughout their differentiation process with C4BP(β+), C4BP(β-) (both at 12 nM) and PRP6-HO7 (32 nM). DC maturation was achieved by TLR agonist treatment: Poly I:C HMW (TLR3), Poly I:C LMW (TLR3), Gardiquimod (TLR7), ssRNA40/LyoVec (TLR8), *E.coli* ssDNA/LyoVec (TLR9). Cells were then collected, washed, and analyzed by flow cytometry for cell surface expression of CD83, CD86, CD80, CD40 and HLA-DR. MFI, median fluorescence intensity for the different surface markers. iDC, untreated, immature DCs; mDC, untreated, TLR-matured DCs. The results shown are the mean ± SD from 4-10 independent donors (*, p < 0.05; **, p < 0.01; ***, p < 0.001; ****, p < 0.0001 compared with mDC).

We assessed whether PRP6-HO7 was able to restrain the expression of different monocyte and DC surface activation markers, including CD83, CD86, CD80, CD40, and HLA-DR when upregulated through human TLR1 to TLR9, which recognize pathogen-associated microbial patterns (PAMPs) and danger-associated molecular patterns (DAMPs), able to stimulate antigen presentation and promote efficient T cell help. C4BP(β+) had no effect on the expression of any of the above markers in DCs matured with the indicated TLR agonists. In contrast, both C4BP(β-) and PRP6-HO7 significantly down-regulated not only CD83 and CD86, as previously stated, but also the co-stimulatory molecules CD80 and CD40, and the MHC class II cell surface receptor HLA-DR on Mo-DCs activated by all extracellular (**Figure 5**) and intracellular (**Figure 6**) TLRs but TLR9, which is not expressed in these cells (26).

These data confirm that PRP6-HO7 has the potential to prevent TLR-induced pro-inflammatory DC differentiation/maturation as judged by the expression pattern of various cell surface markers. In contrast, neither PRP6-HO7 alone nor PRP6-HO7 plus LPS incubated from day 5 to day 7 (maturation) affected DC surface marker expression (data not shown).

### Human Serum Does not Interfere With the PRP6-HO7 Immunomodulatory Activity

To infer the behavior of PRP6-HO7 in a more complex environment we analyzed CD83 and CD86 surface marker expression on DCs differentiated and LPS-matured in presence of 50% autologous serum. Analogously to C4BP(β-), PRP6-HO7 was able to significantly downregulate the above markers in a dose-dependent manner (**Supplementary Figure 1**). Thus, PRP6-HO7 was as active as its counterpart C4BP(β-) in immunomodulation under a cell-free, near to circulatory system state.

### PRP6-HO7 Alters the Chemotaxis and T Cell Alloproliferation Capacities of Mo-DCs

**Figure 7:**
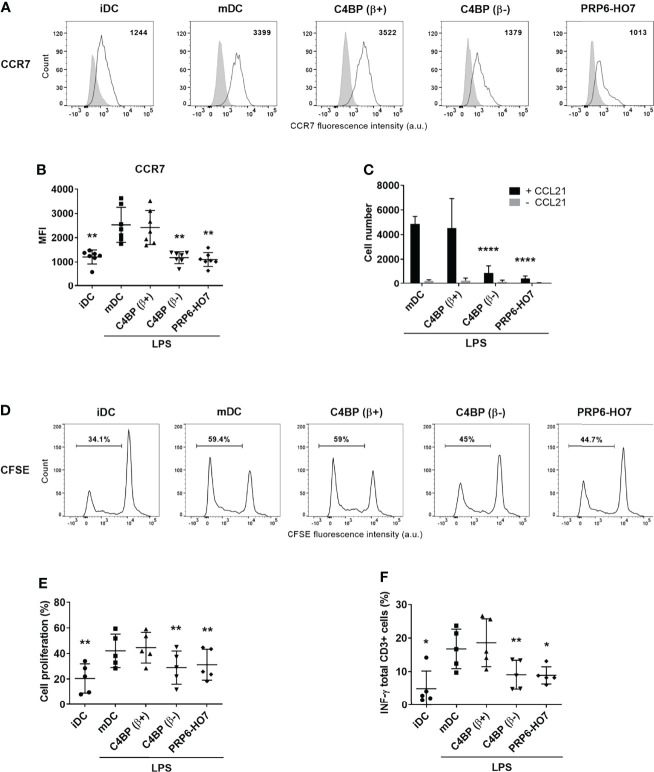
|PRP6-HO7 down-regulates CCR7 expression, alters the chemotaxis, and prevents T cell alloproliferation and IFN-γ production in Mo-DCs. CCR7 expression analysis of Mo-DCs at translational level. Representative histograms displaying CCR7 surface expression **(A)**, and its quantification **(B)** on C4BP(β+)-treated, C4BP(β-)-treated (both at 12 nM), and PRP6-HO7-treated (32 nM) and LPS-matured DCs. Isotype control is shown in gray. The MFIs for CCR7 cell surface expression are indicated. Results shown are the mean ± SD from 7 independent donors. **(C)** Migration of untreated, C4BP(β+)-treated, C4BP(β-)-treated (both at 12 nM), and PRP6-HO7-treated (32 nM) DCs towards the chemokine CCL21 after LPS maturation was assessed in a transwell assay. Shown are the absolute numbers of LPS-matured DCs (mDC) migrated toward the lower CCL21-containing chamber after 2 h incubation (black columns). Spontaneous migration of DCs towards a lower chamber without CCL21 was also assessed (grey columns). Results are the mean ± SD from 7 independent donors performed in duplicate. Allogeneic CD3+ T cells were labeled with the CSFE dye and co-cultured with C4BP(β+)-treated, C4BP(β-)-treated (both at 12 nM), and PRP6-HO7-treated (32 nM) and LPS-matured DCs at 1:5 DC:T cell ratio. **(D)** Histograms from one representative experiment indicating the percentage of proliferating cells that have lost the CSFE dye. **(E)** Quantification of the percentage of T cell proliferation. **(F)** Percentage IFN-γ production by CD3+ T cells. Results shown are the mean ± SD from 5 independent donors. iDC, untreated, immature DCs; mDC, untreated, LPS-matured DCs (*p < 0.05; **p < 0.01; ****p < 0.0001 compared with mDC).

Maturation signals determine the expression of distinct DC functions, such as migration towards lymph node-directing chemokines. Both C4BP(β-) and PRP6-HO7 treatments down-regulated the expression of the chemokine receptor CCR7 (**Figures 7A, B**). Reduced surface CCR7 expression, in turn, significantly decreased the migration of LPS-matured DCs towards the chemokine CCL21 (**Figure 7C**). In contrast, LPS maturation of both untreated and C4BP(β+)-treated DCs induced a substantial migration in response to CCL21.

Tolerogenic Mo-DCs generated through the C4BP(β-) isoform were previously found to impact on the proliferation of allogeneic T cells and their polarization towards a Th1 phenotype (13). Accordingly, we next examined the immunostimulatory capacity of Mo-DCs exposed to PRP6-HO7. When Mo-DCs were pre-incubated with the C4BP(β+) isoform and matured with LPS, maximal allogeneic T cell proliferation was observed, similar to that obtained using untreated, LPS-matured Mo-DCs. In contrast, mature Mo-DCs pre-incubated with PRP6-HO7 prevented CD3^+^ T cell proliferation (**Figures 7D, E**) and IFN-γ production (**Figure 7F**), approaching the levels observed using iDCs. Thus, PRP6-HO7-treated DCs impair T cell alloproliferation.

### PRP6-HO7 Modulates Both TLR-Mediated and Intrinsic Activation in Mo-DCs From Active Autoimmune SLE Patients

SLE is a complex autoimmune disorder characterized by loss of tolerance to self-antigens, increased production of autoantibodies and deposition of complement-fixing immune complexes mainly in the kidneys. As a proof of concept to assess the immunomodulatory potential of PRP6-HO7 in autoimmune pathologies, we undertook pilot studies in two consecutive small cohorts of SLE patients, mostly with active LN, termed cohort 1 and cohort 2. In cohort 1 we examined whether Mo-DCs from active lupus patients responded to PRP6-HO7 immunomodulation. In cohort 2 we explored whether PRP6-HO7 could revert the intrinsic activation state of these cells.

**Figure 8:**
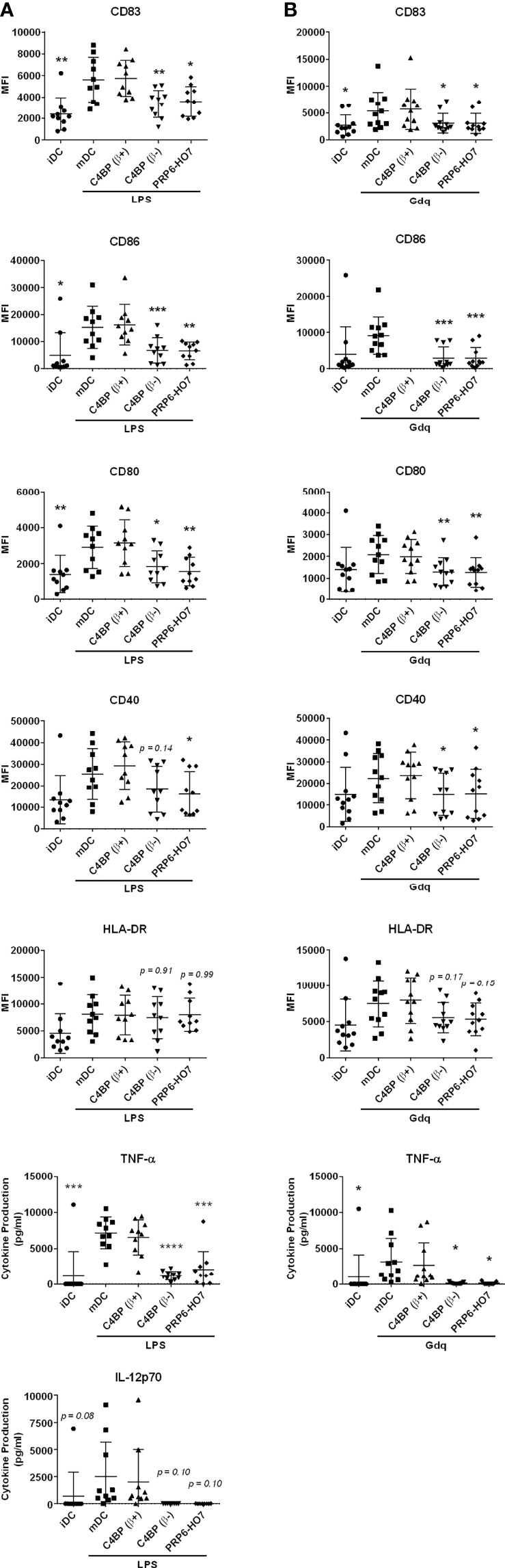
PRP6-HO7 displays immunomodulatory activity in differentiating Mo-DCs and Mo-macrophages from active autoimmune SLE patients. Human Mo-DCs from active SLE patients (cohort 1) were incubated throughout their differentiation process with C4BP(β+) and C4BP(β-) (both at 12 nM), and with PRP6-HO7 at 32 nM. DC maturation was achieved by LPS **(A)** or gardiquimod (Gdq) **(B)** treatment. Cells were then collected, washed, and analyzed by flow cytometry for cell surface expression of the activation marker CD83, the co-stimulatory molecules CD86, CD80 and CD40, and HLA-DR. MFI, median fluorescence intensity for the different surface markers. The concentrations of IL-12p70, and TNF-α were analyzed in the respective cell supernatants by ELISA. The results shown are the mean ± SD from 10 (LPS) or 11 (Gdq) independent donors. iDC, untreated, immature DCs; mDC, untreated, LPS-matured DCs (*p < 0.05; **p < 0.01; ***p < 0.001; ****p < 0.0001 compared with mDC).

The demographic and clinical features of cohort 1 are given in **Supplementary Table 1**. Monocytes isolated from these patients, either untreated or treated with C4BP(β+), C4BP(β-) or PRP6-HO7, were differentiated to Mo-DCs and further matured with the TLR4 ligand LPS or the TLR7 agonist gardiquimod. As previously shown, significant immunomodulatory activity from both C4BP(β-) and PRP6-HO7 was observed over Mo-DCs from healthy individuals when matured with pro-inflammatory LPS or gardiquimod (**Figures 5**, **6**). Analogously, C4BP(β-)- and PRP6-HO7-treated Mo-DCs isolated from active LN patients and matured with LPS or gardiquimod downregulated not only surface activation markers (CD83, CD86, CD80, CD40), but also pro-inflammatory cytokines such as TNF-α and IL-12 (the latter only through LPSactivation, although it did not reach statistical significance) (**Figure 8**).

**Figure 9:**
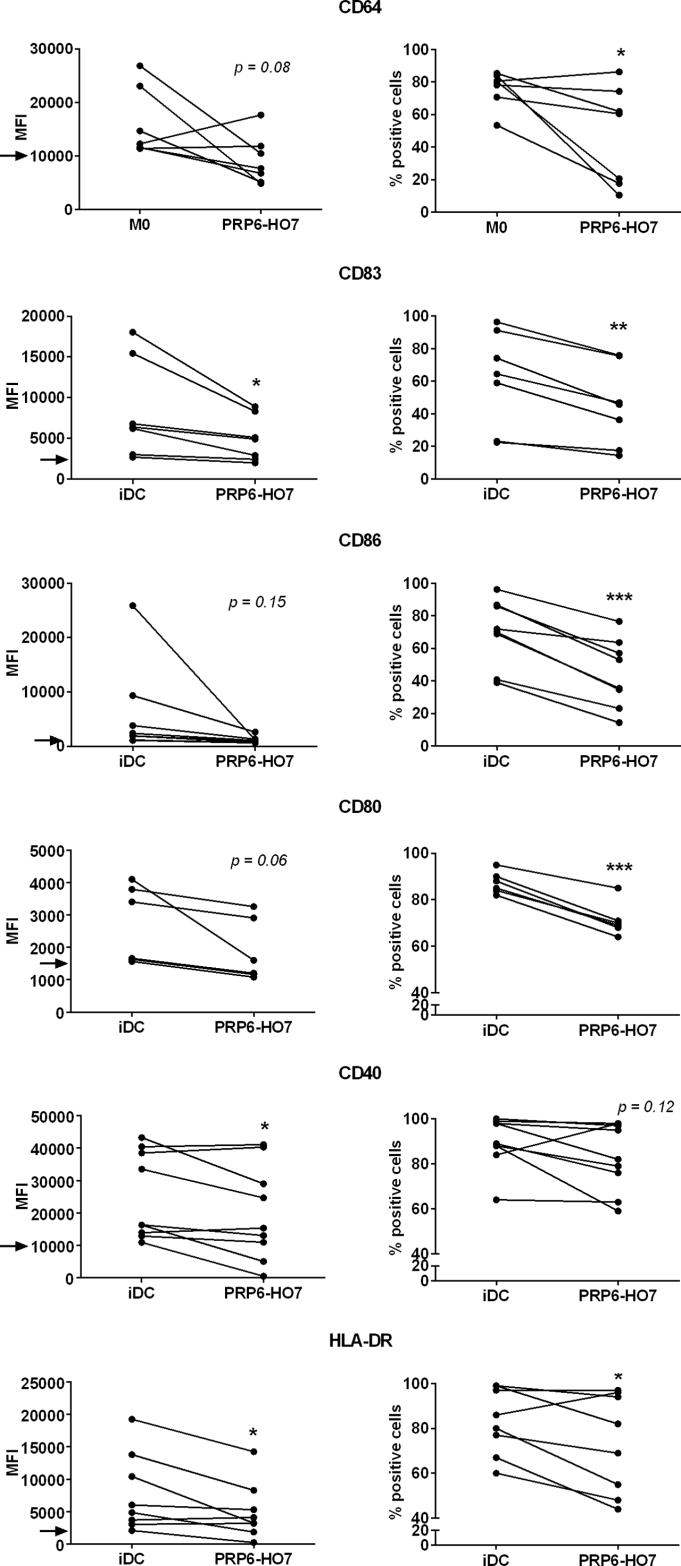
PRP6-HO7 modulates intrinsically activated surface markers in differentiating Mo-DCs and Mo-macrophages from active autoimmune SLE patients. Human Mo-DCs and Mo-macrophages from active SLE patients (cohort 2) were either left untreated or incubated with PRP6-HO7 at 32 nM throughout their differentiation process. Cells were then collected, washed, and analyzed by flow cytometry for cell surface expression of the activation markers CD64 (M0), or CD83, the co-stimulatory molecules CD86, CD80 and CD40, and HLA-DR (iDC). MFI, Both the median fluorescence intensity (MFI) and the percentage of positive cells for the different surface markers are indicated. The arrows indicate the MFI threshold of activity considered for each of the surface markers (CD64: 10000, CD83: 2500, CD86: 1000, CD80: 1500, CD40: 10000, HLA-DR: 2000). The results shown are the mean ± SD from 6-9 independent donors (*p < 0.05; **p < 0.01; ***p < 0.001 compared with M0 or iDC).

We further analyzed in a second cohort of SLE patients (cohort 2), the majority having a LN flare (**Supplementary Table 2**), the intrinsic expression levels of distinctive surface markers on monocyte-derived non-activated macrophages (M0) (CD64) or monocyte-derived immature DCs (iDC) (CD83, CD86, CD80, CD40, and HLA-DR). Although the number of patients analyzed was small, the expression of the above surface markers, reflecting the inflammatory activation state of the cells, appeared to correlate with typical clinical disease activity parameters from LN. Thus, from the available clinical data, complement proteins such as Factor B negatively correlated with CD83, CD86, CD80 and CD40; C3 negatively correlated with CD83; and C4 tended to negatively correlate with CD83 and CD86. Regarding circulating immunoglobulins, IgG levels negatively correlated with CD83 and CD86, and IgA levels negatively correlated with CD80. In contrast, IgM levels positively correlated with CD80. Anti-C1q levels negatively correlated with CD80 and CD40, and anti-nucleosomes levels negatively correlated with CD83. Concerning immune cells, the blood leukocyte number tended to positively correlate with CD86 and CD40 and, particularly, lymphocyte number positively correlated with CD80 and CD40. Platelet number tended to positively correlate with CD80. Finally, biochemical parameters such as serum creatinine negatively correlated with CD80, serum albumin appeared to negatively correlate with both CD64 and CD83, serum ferritin negatively correlated with CD80 and CD40, and the glomerular filtrate positively correlated with CD80. No significant correlations could be observed with HLA-DR (**Supplementary Figure 2**). Considering this outcome, we set up thresholds of activation for each of the surface markers analyzed. Thus, compared to untreated iDC and M0 from the isolated patients’ monocytes, PRP6-HO7 significantly downregulated both the levels and/or the percentage of cells positive for each of the above markers (**Figure 9**).

Together, these data suggest that PRP6-HO7 can immunomodulate inflammatory immune cells from patients with active autoimmune lupus nephritis, regardless of their medical history.

## Discussion

C4BP is a multifunctional protein with well-known roles within the complement system as a cofactor for factor I-mediated C4b inactivation and promotor of C3 convertase decay (27). In addition, its major isoform, the heterooligomer C4BP(β+), bound tightly to PS through its β-chain, interacts with dying cells promoting its silent clearance and, therefore, contributes to cellular and tissue homeostasis by preventing unnecessary inflammation (28). Nevertheless, the ultimate reason for the existence of C4BP isoforms with different α- and β-chain composition and the physiological relevance of the minor C4BP isoforms have not been yet clarified. We identified an additional immunomodulatory activity in the most abundant of the minor C4BP isoforms, the homooligomer C4BP(β-), which is not present in the major C4BP(β+) isoform. This activity was localized in the internal CCP6 domain of the C4BP α-chain (13). Although all circulating C4BP(β+) molecules are bound to PS abolishing its anticoagulant function, in this report we reveal that the C4BP β-chain, unaided by PS, is responsible for the lack of immunomodulatory activity of C4BP(β+). Oligomeric C4BP comprises 60-amino acid CCP or sushi domains arrayed linearly shaping its α- and β-chains and holds a spider-like structure, with the carboxy-terminal ends of the seven CCP8 α-chains and the CCP3 β-chain joined together through the oligomerization domain (29, 30). This modularity endows the different C4BP isoforms with the flexibility to interact with C4b, although each molecule of C4BP can only bind four molecules of C4b due to steric hindrance (31). Analogously, both the more limited flexibility of the internal CCP6 domain of the α-chain and the presence of the β-chain likely impedes C4BP(β+) proper interaction with the corresponding cell surface receptor(s) through steric hindrance, hindering the immunomodulatory activity evidenced in C4BP(β-).

The murine *C4BPB* gene has become a pseudogene in the mouse and, consequently, mouse C4BP lacks the β-chain (32). Strikingly, the α-chain of mouse C4BP also lacks the CCP6 domain (33, 34). Although seemingly unconnected, these two phenomena could be evolutionarily and functionally related. Conceivably, upon C4BP β-chain loss, mice might have been forced to opt out the CCP6 domain of C4BP α-chain to avoid increased susceptibility to pathogen infection. It is well known that pathogens recruit C4BP for protection (28). Thus, upon C4BP binding, pathogens could leverage the strong anti-inflammatory and immunomodulatory activity of its CCP6 domain towards the host’s innate immune cells as an alternative immune evasion strategy.

We further confirmed the independence of both canonical and non-canonical activities of C4BP(β-). In fact, CCP-deletion variant PRP5/8-HO7, devoid of the N-terminal α-chain CCP1-CCP4 domains critical for C4b and C3b binding and complement inhibition (27), retained the immunomodulatory activity over Mo-DCs. Furthermore, PRP6-HO7, a minimal recombinant variant synthesized by joining the immunomodulatory CCP6 α-chain domain and the oligomerization domain of C4BP, was sufficient to confer full immunomodulatory activity, being devoid not only of the canonical complement inhibitory activity but also of detrimental pathogen binding leading to complement immune evasion. Indeed, recruitment of human C4BP onto the surfaces of Gram-positive and Gram-negative bacteria, viruses and fungi has been described (28, 35), rendering the host more susceptible to infection episodes (36). We also demonstrated that the CCP6 domain of the C4BP α-chain needs to be oligomerized, analogously to C4BP(β-), to preserve full immunomodulatory activity. This multivalent structure likely interacts with high avidity on a multivalent receptor or a cluster of monovalent receptors, reminiscent of IgG and IgM molecules. Indeed, the oligomeric, spider-like structure of both C4BP(β-) and PRP6-HO7 suggests that induction of receptor clustering could increase their strength for signaling. In fact, the oligomerization domain of C4BP provides the optimal distance between multiple protein-binding domains to maximize binding affinities to multimeric target receptors *via* multivalent interactions (37, 38) and, consequently, has also been exploited to increase the valency and performance of recombinant antibodies (39), soluble viral receptors (40), apoptosis-inducing peptides (41), or heteromultimeric FHR4-based immunoconjugates selectively activating the complement alternative pathway on tumor cells (42).

Compared with C4BP(β-), oligomeric PRP6-HO7 holds a reduced size (100 kDa) and its compact and symmetrical structure likely impacts positively in its thermodynamic stability and *in vivo* half-life. It has been shown that CCP-containing proteins and, particularly C4BP, hold a remarkable structural and functional stability after being exposed to harsh conditions (43). Moreover, the whole α-helix region constituting the oligomerization domain is required to form stable polymers, which are further stabilized by intermolecular disulfide bond formation and electrostatic interactions (15, 18). Consequently, analogously to C4BP(β-) (13), under a cell-free, near to circulatory system state (50% human serum) PRP6-HO7 displayed full immunomodulatory activity over Mo-DCs, a faithful model of *in vivo* inflammatory DCs (44). Moreover, PRP6-HO7 was able to down-regulate the expression of the maturation marker and immune checkpoint CD83 (45), co-stimulatory and MHC molecules, to abrogate the secretion of pro-inflammatory cytokines and, conversely, to increase the production of anti-inflammatory cytokines upon pro-inflammatory challenge. In addition, PRP6-HO7-treated Mo-DCs also displayed increased endocytosis, reduced CCR7 expression and chemotaxis, and decreased proliferation and IFN-γ production of co-cultured allogeneic T cells. These outcomes are typical of regulatory or tolerogenic DCs, which hold a semi-mature phenotype characterized by clonal T cell anergy induction and/or metabolic T cell modulation, and anti-inflammatory cytokine production, leading to suppression of effector T cell activity and stimulating the differentiation of regulatory T cells (Tregs) (46–48). Interestingly, “PRP6-HO7-reprogrammed” Mo-DCs prevented the upregulation of activation markers, co-stimulatory molecules, and HLA-DR, regardless of the TLR agonist employed, suggesting that PRP6-HO7 is effective against a plethora of PAMPs and DAMPs, modulating common downstream MYD88- and TRIF-dependent pathways (49, 50).

We recently demonstrated the therapeutic potential of human C4BP(β-) in two autoimmune LN mouse models, which prevented pro-inflammatory immune cell infiltration and the development of ectopic lymphoid structures, suggesting that the putative C4BP(β-) receptor is preserved in mice (14). Here, we have extended our studies to Mo-DCs and Mo-macrophages obtained from SLE patients with active disease and renal involvement. We demonstrated that both C4BP(β-) and PRP6-HO7 significantly prevented CD83 and co-stimulatory molecule (CD86, CD80, CD40) upregulation and pro-inflammatory cytokine (TNF-α, IL-12) production in Mo-DCs stimulated with LPS and, particularly, with gardiquimod. This imidazoquinoline is a strong agonist of endosomal TLR7, which has a critical role accelerating disease in SLE (51, 52). Furthermore, in immature Mo-DCs and non-polarized M0 Mo-macrophages differentiated from monocytes isolated after a separate cohort of SLE patients, mostly having an LN flare, PRP6-HO7 again downregulated high-level expression of pro-inflammatory cellular surface molecules such as IFN-I-inducible FcγRI/CD64, a biomarker reflecting ongoing inflammation and nephritis in lupus (53, 54), CD83, CD86, CD80, CD40, and HLA-DR without the need of further stimulation. Indeed, these immune cell markers correlated with the most relevant quantitative serum parameters defining LN activity/severity (complement components, immunoglobulins, immune cells, organic compounds, and proteins (creatinine, albumin, ferritin), and with the glomerular filtrate). For example, renal complement deposition has been proposed as a predictor of end-stage renal disease in LN patients (55). Moreover, autoantibodies and pathogenic immune complex accumulation in multiple tissues are also hallmarks of the disease (56, 57). All these events result in the consumption of circulating complement components and hypogammaglobulinemia, except for IgM, which might bind preferentially circulating autoantigens such as dsDNA. Strikingly, although all patients analyzed presented with ongoing medication (hydroxychloroquine, corticoids, and/or immunosuppressants), these treatments seemed to have no influence on the immunomodulatory efficacy of PRP6-HO7, probably because of its distinctive mechanism of action.

## Conclusion

In summary, the immunomodulatory activity of the minor C4BP(β-) isoform and the specific up-regulation of its expression under strong pro-inflammatory conditions (10, 11, 58) suggests that, besides complement regulation, this isoform exerts an additional level of control from excessive inflammation through a gain of function over the major isoform C4BP(β+), which allows more efficient control of the innate immune system at both the humoral side (complement regulation) and the cellular side (phagocyte immunomodulation). Furthermore, this non-canonical immunomodulatory activity of C4BP(β-) is independent of anticoagulant PS and, therefore, would additionally benefit systemic inflammatory and hypercoagulable conditions such as sepsis (59). Thus, C4BP(β-) directly impacts the cellular innate immune response, acting on pro-inflammatory monocytes, “reprogramming” them from a pro-inflammatory and immunogenic phenotype towards an anti-inflammatory and tolerogenic phenotype, resolving excessive inflammation and “sculpting” the adaptive immune system to circumvent autoimmunity. Accordingly, PRP6-HO7, a C4BP(β-)-based recombinant oligomeric protein, shows a compact structure and is smaller than an antibody in size. Most importantly, PRP6-HO7 displays strong anti-inflammatory and immunomodulatory activities facilitating return to homeostasis and, conversely, lacks complement inhibitory activity and pathogen binding for immune evasion. Thus, its optimal performance and improved safety profile, arising from a natural protection system such as C4BP(β-), supports the development of PRP6-HO7 as a first-in-class biologic for the treatment of SLE and other autoimmune diseases.

## Data Availability Statement

The original contributions presented in the study are included in the article/**Supplementary Material**. Further inquiries can be directed to the corresponding author.

## Ethics Statement

The studies involving human participants were reviewed and approved by the IDIBELL’s ethics committee in accordance with institutional guidelines and the Declaration of Helsinki. The patients/participants provided their written informed consent to participate in this study.

## Author Contributions

JA conceived and designed the study. IS, AL, FM, SR, CV and JT performed experiments and provided key reagents. IS, AL, AB, SR, CV, JT and JA analyzed and interpreted the data. IS, AL and JA wrote the manuscript. All authors contributed to the article and approved the submitted version.

## Funding

We thank CERCA Programme/Generalitat de Catalunya for institutional support. This work was supported by the Ministerio de Ciencia, Innovación y Universidades (Madrid, Spain) (grants FIS-ISCIII PI20/00464, PI16/00377 and DTS20/00016), and the “Departament de Recerca i Universitats de la Generalitat de Catalunya” (grants 2019PROD00081 and 2017SGR291), all co-funded by FEDER funds/European Regional Development Fund (ERDF)-a way to build Europe-. Dr. Vega is supported by the Spanish “Ministerio de Ciencia, Innovación y Universidades/FEDER” [RTI2018-102242-B-I00]. Dr. Rodriguez de Córdoba is supported by the Spanish “Ministerio de Economia y Competitividad/FEDER [SAF2015-66287-R]. Dr. Vega and Dr. Rodriguez de Córdoba are also funded by the Autonomous Region of Madrid [S2017/BMD-3673] and the European Commission – NextGenerationEU through CSIC’s Global Health Platform (“PTI Salud Global”) [SGL2103020]. The funders had no role in the study design, data collection and analysis, decision to publish, or preparation of the manuscript.

## Conflict of Interest

IS, AL and JA are co-inventors on pending or issued patents involving compounds and methods for immunomodulation.

The remaining authors declare that the research was conducted in the absence of any commercial or financial relationships that could be construed as a potential conflict of interest.

## Publisher’s Note

All claims expressed in this article are solely those of the authors and do not necessarily represent those of their affiliated organizations, or those of the publisher, the editors and the reviewers. Any product that may be evaluated in this article, or claim that may be made by its manufacturer, is not guaranteed or endorsed by the publisher.
