# Supplementary material for: The Hidden Side of Complement Regulator C4BP: Dissection and Evaluation of Its Immunomodulatory Activity
Source: Front Immunol. 2022 Apr 25;13:883743. doi: 10.3389/fimmu.2022.883743 (PMC9084231; doi:10.3389/fimmu.2022.883743)
Supplement: Supplementary file 1 [file DataSheet_1.pdf]

Supplementary Material

1 Supplementary Figures and Tables

1.1 Supplementary Figures

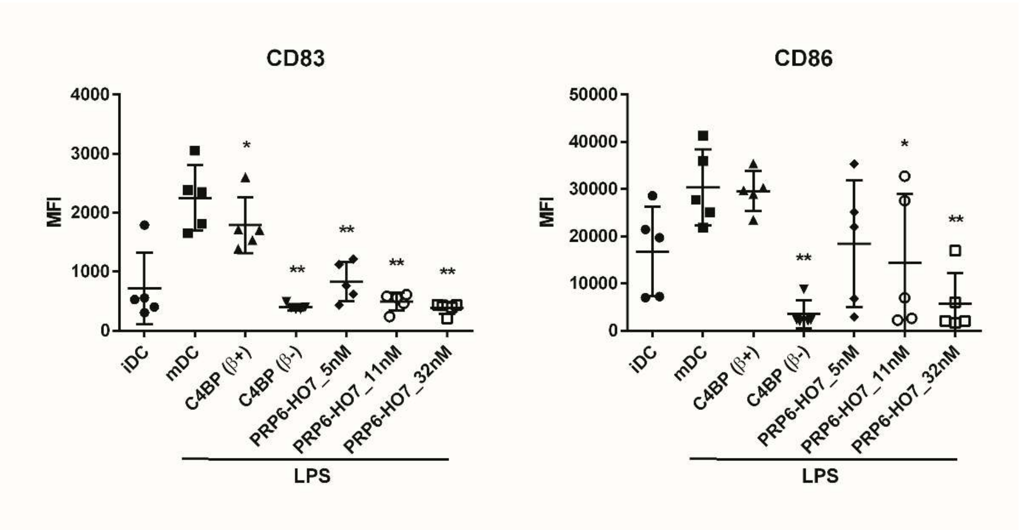

Supplementary Figure 1.- PRP6-HO7 downregulates CD83 and CD86 expression in LPS-activated Mo-DCs grown in 50% human serum.

Human Mo-DCs were grown in RPMI 1640 cell culture medium supplemented with 50% heat-inactivated autologous serum and incubated throughout their differentiation process with C4BP(β+) and C4BP(β-) (both at 12 nM), and with the indicated concentrations of PRP6-HO7. DC maturation was achieved by LPS treatment. Cells were then collected, washed, and analyzed by flow cytometry for cell surface expression of CD83 and CD86. MFI, median fluorescence intensity for the different surface markers. iDC, untreated, immature DCs; mDC, untreated, LPS-matured DCs. The results shown are the mean ± SD from 5 independent donors (*, $p < 0.05$; ** $p < 0.01$ compared with mDC).

(I)

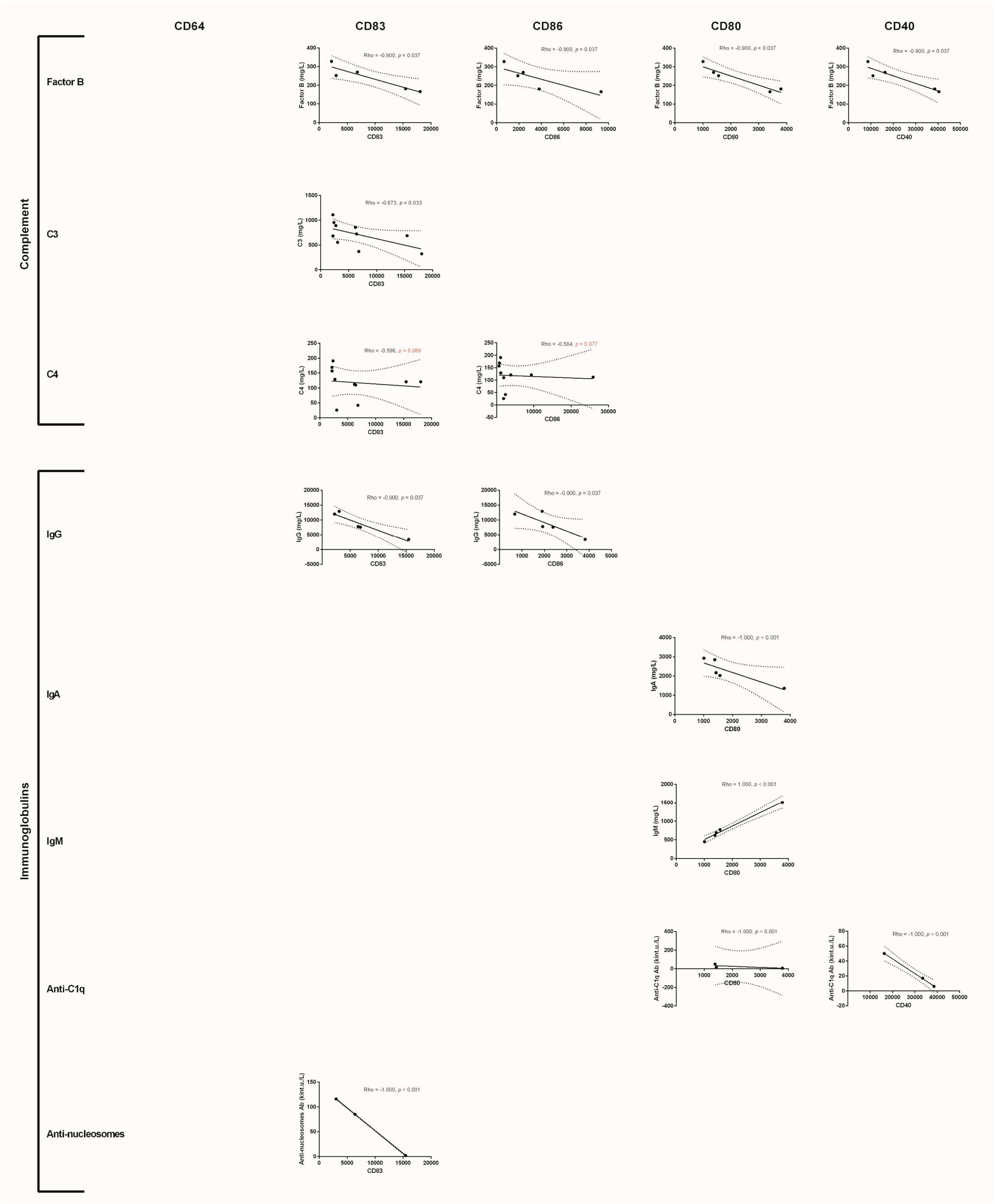
CD64
CD83
CD86
CD80
CD40
Complement
Factor B
C3
C4
Immunoglobulins
IgG
IgA
IgM
Anti-C1q
Anti-nucleosomes
Rho = -0.900, p = 0.037
Rho = -0.673, p = 0.033
Rho = -0.596, p = 0.069
Rho = -0.584, p = 0.077
Rho = -1.000, p < 0.001
Rho = 1.000, p < 0.001
Factor B (mg/L)
C3 (mg/L)
C4 (mg/L)
IgG (mg/L)
IgA (mg/L)
IgM (mg/L)
Anti-C1q Ab (kint.u./L)
Anti-nucleosomes Ab (kint.u./L)

(II)

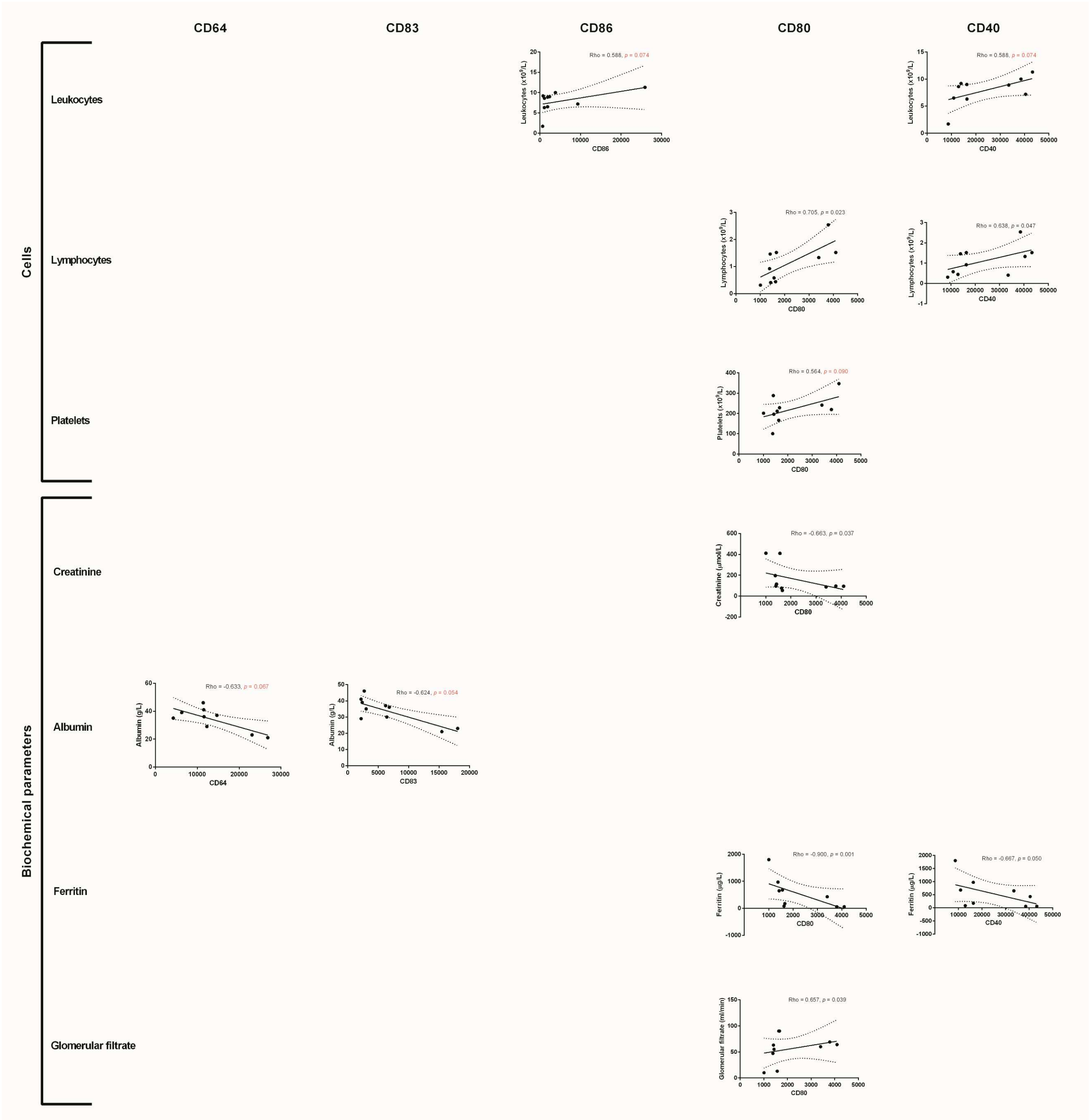

Supplementary Figure 2.- Surface marker expression on Mo-DCs and Mo-macrophages correlate with clinical features of lupus nephritis disease activity in SLE patients.

Matrix-type image showing the correlations found between the surface markers analyzed (CD64, CD83, CD86, CD80, and CD40) and clinical data gathered from cohort 2 SLE patients, including: **(I)** complement components (Factor B, C3, C4) and immunoglobulins (IgG, IgA, IgM, anti-C1q antibodies, anti-nucleosomes antibodies), and **(II)** blood cells (leukocytes, lymphocytes, platelets), and biochemical parameters (serum creatinine, serum albumin, serum ferritin, glomerular filtrate). Both correlation coefficients and p values (in red whether approaching significance) are highlighted for each of the correlations.

1.2 Supplementary Tables

Supplementary Table 1.- Main clinical characteristics of SLE patient cohort 1

Patient characteristics	Total n	mean / n	±SD / %
Age (years)	11	47.3	13.5
Sex (% Female)	11	8	72.7
Smoking	11	6	54.5
Dyslipidemia	11	6	54.5
Hypertension	11	4	36.4
Diabetes mellitus II	11	0	0
SLEDAI	11	16.8	10.2
Lupus nephritis	11	9	81.8
Flare	11	8	72.7
Erythrocytes ($x10^{12}$/L)	11	3.8	0.7
Hemoglobin (g/L)	11	114.2	22.3
Platelets ($x10^{9}$/L)	11	268.5	125.8
Leukocytes ($x10^{9}$/L)	11	6.7	3.2
Lymphocytes ($x10^{9}$/L)	11	0.9	0.5
Monocytes ($x10^{9}$/L)	11	0.5	0.3
Urea (mmol/L)	7	13.4	8.4
Creatinine (µmol/L)	11	160.1	141.4
Glomerular filtration rate (ml/min)	11	59.5	31.7
Albumin (g/L)	11	34.7	7.2
Ferritin (µg/L)	10	475.2	530.2
C- reactive protein (mg/L)	11	17.2	32.6
Proteinuria (g/24h)	7	1.9	2.4
Protein/Creatinine (g/mol)	3	347.5	330
Hematuria (erythrocytes/µl)	8	191.9	508.1
IgG (mg/L)	4	10460	2394.7
IgA (mg/L)	4	2931.3	1179.7
IgM (mg/L)	4	547	228.6
C3 (mg/L)	11	736	242.5
C4 (mg/L)	11	107.6	57.4
Factor B (mg/L)	3	273	48.1

Anti ss-DNA Ab (kint.u./L)	11	417.2	610.9
Anti-nucleosomes Ab (kint.u./L)	4	92.5	51
Anti-C1q Ab (kint.u./L)	2	9.5	10.6
Anti-SS-A Ab (Ro60) (CU)	6	239.8	556.2
Anti-SS-A Ab (Ro52) (CU)	6	49.8	75.9
Anti-SS-B Ab (La) (CU)	5	3.3	0
Anti-RNP Ab (U1) (CU)	6	51.6	112.5
Anti-Sm Ab (CLIA) (CU)	6	14.4	18
Immunosuppressant treatment	11	7	63.6
Hydroxychloroquine treatment	11	6	54.5
JAK Inh treatment	11	0	0
BAFF Inh treatment	11	2	18.2
Steroid/Corticoid treatment	11	8	72.7
Vitamin D treatment	11	4	36.4

Supplementary Table 2.- Main clinical characteristics of SLE patient cohort 2

Patient characteristics	Total n	mean / n	±SD / %
Age (years)	10	48.8	14.8
Sex (% Female)	10	7	70
Smoking	10	4	40
Dyslipidemia	10	6	60
Hypertension	10	3	30
Diabetes mellitus II	10	1	10
SLEDAI	10	19	10.5
Lupus nephritis	10	8	80
Flare	10	8	80
Erythrocytes ($x10^{12}/L$)	10	3.8	0.6
Hemoglobin (g/L)	10	115.9	17
Platelets ($x10^{9}/L$)	10	219.7	66.3
Leukocytes ($x10^{9}/L$)	10	7.9	2.7
Lymphocytes ($x10^{9}/L$)	10	1.1	0.7
Monocytes ($x10^{9}/L$)	10	0.6	0.3
Urea (mmol/L)	7	11.5	5.9
Creatinine (μmol/L)	10	163.7	134.9
Glomerular filtration rate (ml/min)	10	56.1	27.2
Albumin (g/L)	10	33.7	7.9
Ferritin (μg/L)	9	544.5	573.3
C- reactive protein (mg/L)	10	14.5	34.4
Proteinuria (g/24h)	5	0.7	0.6
Protein/Creatinine (g/mol)	5	308.1	259.4
Hematuria (erythrocytes/μl)	8	232.9	495.2
IgG (mg/L)	5	8741	3833.3
IgA (mg/L)	5	2266.6	643.7
IgM (mg/L)	5	809.4	408.1
C3 (mg/L)	10	717.1	249.8
C4 (mg/L)	10	117.8	51.6
Factor B (mg/L)	5	239.4	66.6
Anti ss-DNA Ab (kint.u./L)	10	98.1	102.6
Anti-nucleosomes Ab (kint.u./L)	3	67.7	58.9
Anti-C1q Ab (kint.u./L)	3	24.3	22.9
Anti-SS-A Ab (Ro60) (CU)	6	236.8	557.6
Anti-SS-A Ab (Ro52) (CU)	6	55.8	72.5
Anti-SS-B Ab (La) (CU)	5	3.3	0
Anti-RNP Ab (U1) (CU)	6	49.8	113.3
Anti-Sm Ab (CLIA) (CU)	6	3.3	0

Immunosuppressant treatment	10	5	50
Hydroxychloroquine treatment	10	6	60
JAK Inh treatment	10	1	10
BAFF Inh treatment	10	0	0
Steroid/Corticoid treatment	10	8	80
Vitamin D treatment	10	3	30
